# Association of maternal age with child health: A Japanese longitudinal study

**DOI:** 10.1371/journal.pone.0172544

**Published:** 2017-02-24

**Authors:** Tsuguhiko Kato, Takashi Yorifuji, Michiyo Yamakawa, Sachiko Inoue, Hiroyuki Doi, Akira Eboshida, Ichiro Kawachi

**Affiliations:** 1 Department of Social Medicine, National Center for Child Health and Development, Tokyo, Japan; 2 Department of Human Ecology, Okayama University Graduate School of Environmental and Life Science, Okayama, Japan; 3 Center for Regional Research, Okayama University, Okayama, Japan; 4 Department of Nursing, Okayama Prefectural University Graduate School of Health and Welfare Science, Okayama, Japan; 5 Department of Epidemiology, Okayama University Graduate School of Medicine, Dentistry and Pharmaceutical Sciences, Okayama, Japan; 6 Department of Public Health and Health Policy, Institute of Biomedical Sciences, Hiroshima University, Hiroshima, Japan; 7 Department of Social and Behavioral Sciences, Harvard T.H. Chan School of Public Health, Boston, MA, United States of America; Centre Hospitalier Universitaire Vaudois, FRANCE

## Abstract

Average maternal age at birth has been rising steadily in Western and some Asian countries. Older maternal age has been associated with adverse pregnancy and birth outcomes; however, studies on the relationship between maternal age and young children’s health remain scarce. Therefore, we sought to investigate the association of maternal age with child health outcomes in the Japanese population. We analyzed data from two birth cohorts of the nationwide Japanese Longitudinal Survey of Babies in 21^st^ Century (*n*_2001_ = 47,715 and *n*_2010_ = 38,554). We estimated risks of unintentional injuries and hospital admissions at 18 and 66 months according to maternal age, controlling for the following potential confounders: parental education; maternal parity, smoking status, and employment status; household income; paternal age, and sex of the child. We also included the following as potential mediators: preterm births and birthweight. We observed a decreasing trend in the risks of children’s unintentional injuries and hospital admissions at 18 months according to maternal age in both cohorts. In the 2001 cohort, compared to mothers <25 years, odds ratios of hospital admission at 18 months were 0.97 [95% CI: 0.86, 1.09], 0.92 [0.81, 1.05], 0.76 [0.65, 0.90], and 0.71 [0.51, 0.98] for mothers aged 25.0–29.9, 30.0–34.9, 35.0–39.9, and >40.0 years, respectively, controlling for confounders. Our findings were in line with previous findings from population-based studies conducted in the United Kingdom and Canada suggesting that older maternal age may be beneficial for early child health.

## Introduction

Among developed countries, average maternal age at first birth has been rising steadily in recent decades [1]. In countries such as the United Kingdom (UK), Germany, and Korea, average maternal age at first birth is around 30. In England and Wales, the fertility rate from 1981 to 2012 decreased by about 30% among women aged between 20 and 24, but has doubled among women above 35 [2]. In Japan, average maternal age at first birth increased from 24.4 in 1950 to 30.3 in 2013 [3].

From an obstetric perspective, older maternal age is associated with increased risks of adverse pregnancy and birth outcomes. For example, risks of gestational diabetes and pre-eclampsia both increase with maternal age, as well as fetal risks of low birthweight, preterm births, miscarriage, and congenital anomalies [4–7]. However, studies on the potential risks of advanced maternal age on children’s later health outcomes have remained scarce.

To our knowledge, only a few population-based studies have explored the relationship between maternal age and children’s health. In a UK-based study, Sutcliffe et al. examined the associations of maternal age with child health and development at 9 months, 3 years, and 5 years of age [8]. They found that older maternal age was generally associated with better health (i.e., unintentional injuries and hospital admissions) after controlling for various factors, including income, maternal education, and paternal age.

Another population-based study was conducted in Canada using the National Longitudinal Survey of Children and Youth [9]. Researchers found that children’s general health seemed to improve with older maternal age. However, differences in child health across maternal age groups were not statistically significant once socioeconomic factors were considered.

The findings from the UK and Canada have been suggestive, but no similar studies have yet been undertaken with Asian populations. The rise in average maternal age has also been observed in East Asian countries such as Japan and Korea, but social and cultural context differs greatly from Western countries [3, 10]. Therefore, we sought to examine the association between maternal age and children’s general health outcomes in Japan using the nationwide Longitudinal Survey of Babies in 21^st^ Century. Based on the UK study findings, we hypothesized that older maternal age is associated with better child health.

## Methods

The Longitudinal Survey of Babies in 21^st^ Century (LSB21) is an ongoing study in Japan comprising two birth cohorts, in which the first started in 2001 and the second in 2010. The main purpose of the LSB21 is for the Ministry of Health, Labour and Welfare (MHLW) to develop strategies against the decline of fertility among Japanese families [11]. Details of the LSB21 have been described elsewhere, but the 2010 cohort has not been analyzed [12–14].

**Fig 1:**
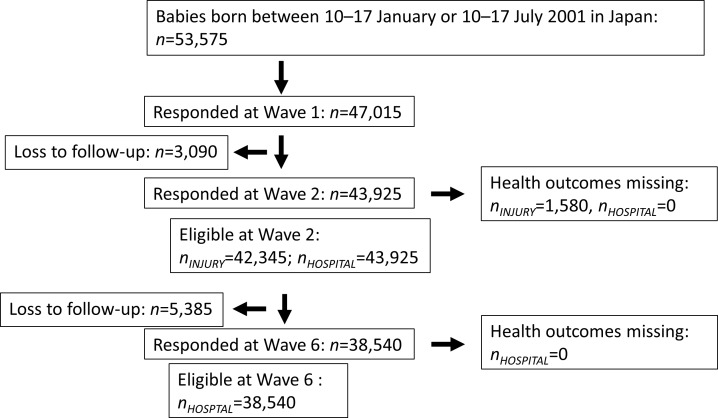
Flow chart of participants from the 2001 cohort. This flow chart shows the number of participants at recruitment, baseline, and each wave, as well as the number of losses at each wave and missing responses within each outcome for the 2001 cohort.

**Fig 2:**
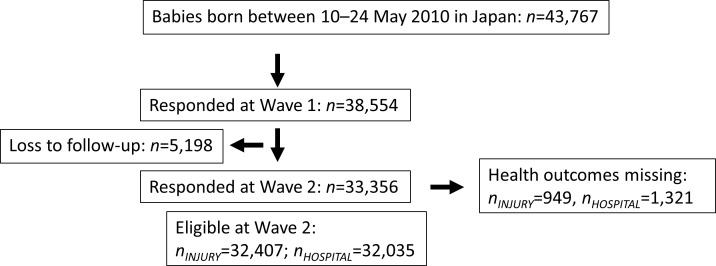
Flow chart of participants from the 2010 cohort. This flow chart shows the number of participants at recruitment, baseline, and each wave, as well as the number of losses at each wave and missing responses within each outcome for the 2010 cohort.

Families from all over Japan were considered eligible to participate in the survey if their child was born between the 10^th^ and 17^th^ of January or July in 2001 or between the 10^th^ and 24^th^ of May in 2010. Baseline questionnaires were sent to all families when infants reached 6 months of age (cf. Wave 1). For both cohorts, the response rates were 88%. Follow-up questionnaires were sent to participating families every 12 months, at 18 months (Wave 2), 30 months (Wave 3), and so on. Birth record information included birthweight, gestational age, sex of the child, and parental age. We obtained information on Waves 1–12 (the 2001 cohort) and Waves 1–3 (the 2010 cohort) from the MHLW. Participant numbers and loss to follow-up are summarized in Figs 1 and 2. This study was approved by the Japanese National Center for Child Health and Development Institutional Review Board (No. 1007).

## Maternal age

For both cohorts, we obtained parental age at delivery from their children’s birth records. We classified maternal age into five categories: 1) younger than 25.0 years; 2) 25.0–29.9 years; 3) 30.0–34.9 years; 4) 35.0–39.9 years, and 5) 40.0 years and older. The Japan Society of Obstetrics and Gynecology considers 35 years or older as advanced maternal age for first-time births [15].

### Unintentional injuries and hospital admissions

In both cohorts, children’s history of unintentional injuries was ascertained from parents at Wave 2, or at the child’s age of 18 months, with the question: “Did your child experience unintentional injuries during the past 12 months?”. Respondents who confirmed their child’s experience of unintentional injuries were then asked to select the type(s) of injury from the following 10 options: 1) a fall from bed, stairs, etc; 2) trapped an arm or a leg between doors or windows; 3) cut with a sharp object such as a knife; 4) bitten by an animal or stung by a bee; 5) drowned or near-drowned; 6) swallowed a small object, such as a coin or cigarette; 7) inserted a small object into the eye, ear, or nose; 8) burnt by a hot object such as a hot iron; 9) had a traffic accident, and 10) other injury. Children’s history of hospital admissions was also ascertained from the parents at Wave 2 with the question: “Was your child admitted to hospital during the past 12 months?”. We assessed the injury outcome at Wave 2 and hospital admission outcomes at Waves 2 and 6 in the 2001 cohort (representing 18- and 66-month longitudinal follow-ups). Similarly, in the 2010 cohort, we assessed injury and hospital admission outcomes at Wave 2. The wording of ascertainment on unintentional injuries and hospital admissions remained exactly the same from the 2001-cohort survey to the 2010-cohort survey.

### Covariate selection

For both cohorts, we considered the same set of socioeconomic and biological factors as potential confounders: maternal and paternal education, maternal smoking status, maternal employment status, household income, sex of the child, maternal parity, and paternal age. We first did not include preterm births and birthweight in our statistical model because these factors are likely to be on the causal pathway between maternal age and child health (i.e. they may be considered intermediary variables) [16]. In the follow-up analyses, however, we additionally included preterm births and birthweight in our analyses to account for their potentially mediating effects. We also examined whether maternal parity (i.e., the experience of having more children) influences the relationship between maternal age and child health via increased knowledge of infant care.

### Variable coding

We obtained data on paternal age, sex of the child, and parity from birth records, maternal smoking status and household income from the first survey, and maternal and paternal education from the second survey. Paternal age was categorized in the same way as maternal age categories. Smoking status was dichotomized as smokers versus non-smokers. We aggregated educational attainment into three levels: completed high school or less, completed two years of college or vocational school, and completed four years of college or more. At Wave 1, questions about maternal employment status one year prior to delivery included seven options ranging from unemployed to full-time employment. We dichotomized these responses into “employed full-time” versus “not employed full-time”. Household income is the combined amount of annual earnings from the father, mother, and other sources, for which JPY ¥10,000 is equivalent to approximately USD $100. Based on gestational age, we created three groups following the WHO classification [17]: term births (>37 weeks), moderately preterm births (32–36 weeks), and very preterm births (<32 weeks). We treated birthweight as continuous.

### Statistical analyses

We conducted logistic regression analyses to evaluate the relationship between maternal age and child health. We first estimated the crude odds ratios (ORs) and 95% confidence intervals (95% CIs) linking the maternal age groups with each health outcome [Unadjusted Model]. We re-calculated the ORs and 95% CIs controlling for potential confounders [Adjusted Model]. In the follow-up analyses, we added preterm births and birthweight to the list of factors included in the adjusted Model [Mediator-adjusted Model]. We also tested whether the associations between maternal age and child health outcomes were linear. In the follow-up analyses, we divided the sample into parity 1 and parity 2 and analyzed the sub-samples using the same models we used in the main analyses. The youngest maternal age group (i.e., <25) served as the reference group, and we conducted our analyses with complete cases. All statistical analyses were performed with Stata (Version 13/SE, STATA Corp., Texas, USA).

## Results

### Baseline characteristics

Average maternal age was 29.9 years in the 2001 cohort (*n* = 47,015) and 31.4 years in the 2010 cohort (*n* = 38,554), which reflects the secular trend toward later childbirth. In Table 1 and S1 Table, we present the distributions of background information in the 2001 and 2010 cohorts by maternal age groups: 25.0–29.9, 30.0–34.9, 35.0–39.9, and >40.0 years. As expected, preterm births and low birthweight were more common among the advanced maternal age groups in both cohorts. The proportions of preterm birth and low birthweight were slightly higher in the <25.0 years group compared to the 25.0–29.9 group in both cohorts, which is likely because of disadvantaged socioeconomic conditions [18]. However, government programs such as national health care coverage to support pregnant women may have been protective against adverse birth outcomes among the youngest mothers in Japan. We also observed that educational attainment and family income tended to be higher among older age groups, but full-time employment was more common among younger mothers. This reflects the pattern of labor force participation among Japanese women, who typically leave work once they begin childbearing [19]. As shown in Figs 1 and 2, we observed some drop-out between the first and subsequent surveys. Those who were lost to follow-up tended to be younger in maternal age with low parental education, maternal smoking habits, and lower household income.

**Table 1:** Baseline characteristics of children by maternal age group in the 2001 cohort of the Longitudinal Survey of Babies in 21^st^ Century (*n*_baseline_ = 47,015).

	<25.0	25.0–29.9	30.0–34.9	35.0–39.9	> = 40.0	*p*-value^b^
	(*n* = 6,327)	(*n* = 18,065)	(*n* = 16,561)	(*n* = 5,406)	(*n* = 656)	
*Biological factors*						
Female	48.8%	47.5%	48.3%	48.4%	46.7%	*p* = 0.36
Birthweight in grams^a^	3026	3029	3042	3046	2996	*p*<0.01
(*SD*)	414	415	440	472	500	
Low birthweight	8.4%	8.1%	8.6%	9.4%	13.3%	*p*<0.01
Preterm birth ^a^	4.8%	4.5%	5.3%	6.2%	9.5%	*p*<0.01
Parity						*p*<0.01
1	76.4%	58.3%	35.9%	27.3%	30.3%	
2	21.3%	34.0%	44.0%	39.6%	32.8%	
3> =	2.3%	7.8%	20.2%	33.2%	36.9%	
*Socioeconomic factors*						
Maternal educational attainment^a^						*p<*0.01
High school or less	67.4%	43.8%	39.8%	40.6%	42.3%	
Two years of college	29.1%	43.1%	43.5%	42.1%	40.0%	
or vocational school						
Four years of college or	3.6%	13.1%	16.8%	17.4%	17.6%	
higher						
Paternal educational attainment^a^						*p<*0.01
High school or less	71.3%	49.8%	41.2%	40.2%	45.2%	
Two years of college	15.2%	17.1%	15.4%	13.0%	10.8%	
or vocational school						
Four years of college or	13.6%	33.2%	43.4%	44.8%	44.0%	
higher						
Maternal employment status^a^						*p<*0.01
Employed full-time	37.0%	36.2%	27.5%	26.4%	25.4%	
Maternal smoking status^a^						*p*<0.01
Smoker	34.5%	18.5%	12.2%	11.1%	13.1%	
Paternal age^a^	25.8	30.2	34.1	38.3	42.4	*p*<0.01
(*SD*)	4.5	4	4.1	4.7	5.1	
Family income^a^	378	526	616	687	713	*p*<0.01
in JPY10,000 (*SD*)	380	317	358	461	550	
at Wave 1						

^a^ 14 missing cases for birthweight, 36 missing cases for preterm birth, 298 missing cases for maternal educational attainment, 792 missing cases for paternal educational attainment, 442 missing cases for maternal employment status, 278 missing cases for maternal smoking status, 612 missing cases for paternal age, and 3194 missing cases for family income.

^b^ We assessed the associations between all biological and socioeconomic factors and maternal age using chi square tests and ANOVA.

### Unintentional injuries and hospital admissions

In Table 2, we present unadjusted and adjusted ORs with 95% CIs for child health outcomes in the 2001 and 2010 cohorts. When children were at the age of 18 months, we observed a linear relationship between older maternal age and lower risks of both unintentional injuries and hospital admission. Even after controlling for potential confounders such as educational attainment of parents, the favorable outcomes remained statistically significant among older maternal age groups compared to the youngest maternal age group. For example, compared to mothers <25 years, ORs of hospital admission were 0.76 [95% CI: 0.65, 0.90] for mothers aged 35.0–39.9 and 0.71 [0.51, 0.98] for >40.0 years, respectively. We statistically confirmed a linear trend by maternal age: *p*_trend_<0.01 for both unintentional injuries and hospital admission. Lower risks among older mothers and the linear relationship between maternal age and child health remained after adjusting for preterm births and birthweight [the Mediator-adjusted Model in the table]. When children were at the age of 66 months, the risks of hospital admission were lower among older maternal age groups. However, the associations attenuated toward null once we controlled for potential confounders.

**Table 2:** Unadjusted, adjusted, and mediator-adjusted odds ratios with 95% confidence intervals and trend test results for associations of maternal age with unintentional injuries at 18 months of child age and hospital admissions at 18 and 66 months in the 2001 cohort (*n*_2001_ = 47,015) and with unintentional injuries and hospital admissions at 18 months in the 2010 cohort (*n*_2010_ = 38,554).

	Unadjusted model	Adjusted model^a^	Mediator-adjusted	*p*_trend_
			model^b^	
2001 Cohort				
Unintentional injuries				
at 18 months^c^				
<25.0	1.00 [reference]	1.00 [reference]	1.00 [reference]	*p*<0.01
25.0–29.9	0.93 [0.86, 1.01]	0.87 [0.78, 0.96]	0.86 [0.78, 0.96]	
30.0–34.9	0.90 [0.83, 0.97]	0.81 [0.73, 0.91]	0.81 [0.73, 0.91]	
35.0–39.9	0.78 [0.70, 0.85]	0.69 [0.61, 0.79]	0.69 [0.61, 0.79]	
> = 40.0	0.72 [0.59, 0.87]	0.66 [0.52, 0.83]	0.65 [0.51, 0.83]	
Hospital admission				
at 18 months				
<25.0	1.00 [reference]	1.00 [reference]	1.00 [reference]	*p*<0.01
25.0–29.9	0.97 [0.89, 1.06]	0.97 [0.86, 1.09]	0.97 [0.86, 1.09]	
30.0–34.9	0.95 [0.86, 1.04]	0.92 [0.81, 1.05]	0.92 [0.80, 1.05]	
35.0–39.9	0.81 [0.72, 0.91]	0.76 [0.65, 0.90]	0.76 [0.64, 0.89]	
> = 40.0	0.80 [0.62, 1.05]	0.71 [0.51, 0.98]	0.70 [0.50, 0.96]	
Hospital admission				
at 66 months				
<25.0	1.00 [reference]	1.00 [reference]	1.00 [reference]	*p* = 0.38
25.0–29.9	0.82 [0.71, 0.95]	0.91 [0.75, 1.10]	0.91 [0.75, 1.10]	
30.0–34.9	0.81 [0.69, 0.93]	0.99 [0.80, 1.22]	0.98 [0.79, 1.21]	
35.0–39.9	0.77 [0.64, 0.93]	1.03 [0.80, 1.34]	1.02 [0.79, 1.32]	
> = 40.0	0.85 [0.57, 1.26]	1.16 [0.73, 1.84]	1.12 [0.70, 1.79]	
2010 Cohort				
Unintentional injuries				
at 18 months				
<25.0	1.00 [reference]	1.00 [reference]	1.00 [reference]	*p*<0.01
25.0–29.9	1.01 [0.91, 1.12]	0.79 [0.69, 0.91]	0.79 [0.69, 0.91]	
30.0–34.9	0.96 [0.87, 1.07]	0.71 [0.61, 0.82]	0.71 [0.61, 0.82]	
35.0–39.9	0.88 [0.79, 0.98]	0.67 [0.57, 0.78]	0.67 [0.57, 0.78]	
> = 40.0	0.81 [0.69, 0.96]	0.63 [0.51, 0.78]	0.64 [0.52, 0.79]	
Hospital admission				
at 18 months				
<25.0	1.00 [reference]	1.00 [reference]	1.00 [reference]	*p* = 0.05
25.0–29.9	0.96 [0.84, 1.09]	0.95 [0.80, 1.14]	0.95 [0.80, 1.14]	
30.0–34.9	0.95 [0.84, 1.08]	0.95 [0.78, 1.14]	0.94 [0.78, 1.14]	
35.0–39.9	0.89 [0.77, 1.02]	0.87 [0.70, 1.06]	0.85 [0.69, 1.05]	
> = 40.0	0.82 [0.65, 1.03]	0.84 [0.63, 1.12]	0.79 [0.59, 1.06]	

^a^ Adjusted for maternal and paternal education, maternal smoking status, maternal employment status one year prior to delivery (employed full-time or not), household income, sex of the child, maternal parity, and paternal age.

^b^ Adjusted for preterm births and birthweight in addition to confounders included in the adjusted model.

^c^ Falls had the highest prevalence with 61%, and the second highest was a trapped arm or leg with 45%. Drowned or near-drowning was the third highest with 12%, while the others were less than 10%. The trend was similar in the 2010 cohort.

In the 2010 cohort, also presented in Table 2, we observed the same trend of better child health at the age of 18 months according to older maternal age as we observed in the 2001 cohort: *p*_trend_<0.01 for unintentional injuries and *p*_trend_ = 0.05 for hospital admission. Although the lower risk for hospital admission among older maternal age groups was not statistically significant, the odds ratios were highly consistent between the 2001 and 2010 cohorts. We did not observe much change in the odds ratios after additionally accounting for preterm births and birthweight. In the follow-up analyses, we stratified the sample by maternal parity. Examining the odds ratios obtained with the sub-samples of parity 1 and parity 2, we did not see much deviation from the original analyses in the magnitude and direction of associations. We present the results in S2 Table.

In Japan, gendered division of labor has been the norm. Therefore, it is predominantly the mothers who take care of their infants. We confirmed this trend in our survey with the question: “who is the child’s main caregiver during the day?”. In the first survey when the child was aged 6 months, we confirmed that 91% of the 2001 cohort and 95% of the 2010 cohort identified ‘mother’ as the main caregiver. We reanalyzed the data with the restricted sample, but we did not see differences from the original analyses.

## Discussion

This is the first population-based study to examine the effect of maternal age on early child health in Asian countries, where the rise in average maternal age at birth has become a serious concern [20]. We found decreased risks of unintentional injuries and hospital admission at 18 months’ follow-up according to older maternal age in both the 2001 and 2010 cohorts. These trends seemed to be robust regarding the adjustment of detailed socioeconomic circumstances and potentially mediating factors such as preterm births and birthweight. We did not observe marked differences in hospital admission at 66 months once we controlled for biological and socioeconomic factors in the 2001 cohort. A key strength of our study is the use of a nationally representative sample of Japanese children from two large birth cohorts. Results were consistent between the two birth cohorts despite the 9-year gap, strengthening the validity of our findings.

Previous studies have revealed that advanced maternal age is a major risk factor for various adverse obstetric and perinatal outcomes [4–6, 21, 22]. Indeed, we observed higher rates of preterm births and low birthweight among older mothers in LSB21. Our previous study, as well as other studies, has shown that preterm birth is a risk factor for unfavorable health outcomes during subsequent years [12, 23]. However, from a socioeconomic perspective, advanced maternal age may also exert a positive impact on children’s health. Japanese women are no exception to the worldwide pattern in which delayed childbearing is associated with higher educational attainment and career investment [24]. In LSB21, women of advanced maternal age tended to have higher educational attainment and higher family income. Evidence suggests that higher socioeconomic status (e.g., higher income and educational attainment) is beneficial for child health [25, 26]. Consequently, social advantage associated with older maternal age may compensate for biological disadvantage [27]. Conversely, when socioeconomic disadvantage threatens maternal “fitness”, there may be an advantage to commencing childbearing at an earlier age—the so-called “weathering hypothesis” proposed by Geronimus [28].

Motherhood readiness linked with age may explain our findings regarding unintentional injuries [29]. Older mothers may be more proactive concerning their children’s exposure to environmental risks of injury. They may also have higher health literacy in managing child illness, thereby avoiding hospitalization compared to younger mothers.

### Comparison with other studies

Our results aligned with those from the UK study by Sutcliffe et al., which showed that advanced maternal age was associated with fewer hospital admissions at 9 months and 3 years and unintentional injuries at 9 months, 3 years, and 5 years [8]. No statistical differences in the risk of hospital admission at 66 months among maternal age groups in our study also corroborated the null association at 5 years in the UK study. Null findings at age 5 in the present study and the UK study may suggest that the influence of other factors such as socioeconomic circumstances intensify with age. The Canadian study showed a similar trend, though the results were not statistically significant [9]. However, the sample size was much smaller (*n* = 3,382) compared to our study and that of Sutcliffe et al. Although social context varies greatly among Japan, the UK, and Canada, results from all three population-based studies seemed to be in agreement.

### Limitations

This study has several limitations. Firstly, inadequate statistical adjustment (i.e., over- or under-adjustment) is always possible. Bias resulting from unmeasured or residual confounding may be present, especially given the complex set of social and biological factors that are correlated with maternal childbearing age. Loss to follow-up occurred in the later waves, particularly among women with a low household income, no job, smoking habits, and women of young maternal age. As these characteristics are often considered as risk factors for child health, the observed prevalence of outcomes within younger maternal age categories may have been underestimated. Recall bias from parental reporting of unintentional injuries and hospitalization admission during the past 12 months seems unlikely because the assessment is rather objective in nature.

## Conclusions

Our results were comparable with those from UK and Canadian populations that suggested associations of older maternal age with improved child health outcomes in early childhood. Although delayed childbearing is an ongoing trend in East Asian countries and globally, its long-term consequences remain poorly understood. Therefore, our study adds to this scarce body of knowledge. However, more studies are needed to validate our findings in different cultural contexts and also explore the effect of older maternal and paternal age on other aspects, such as children’s cognitive and social development.

## Supporting information

S1 Table Baseline characteristics of children by maternal age group in the 2010 cohort of the Longitudinal Survey of Babies in 21st Century (*n*_baseline_ = 38,554). (DOC)

S2 Table Unadjusted, adjusted, and mediator-adjusted odds ratios with 95% confidence intervals and trend test results for associations of maternal age with unintentional injuries at 18 months of child age and hospital admissions at 18 and 66 months in the 2001 cohort (*n*_parity1_ = 22,967 and *n*_parity2_ = 17,119) and with unintentional injuries and hospital admissions at 18 months in the 2010 cohort (*n*_parity1_ = 18,079 and *n*_parity2_ = 14,365), stratified by maternal parity 1 and 2. (DOC)
