# Supplementary material for: Association of maternal age with child health: A Japanese longitudinal study
Source: PLoS One. 2017 Feb 24;12(2):e0172544. doi: 10.1371/journal.pone.0172544 (PMC5325269; doi:10.1371/journal.pone.0172544)
Supplement: S1 Table — (DOC) [file pone.0172544.s001.doc]

S1 Table. Baseline characteristics of children by maternal age group in the 2010 cohort of the Longitudinal Survey of Babies in 21^st^ Century (*n*_baseline_=38,554).

**<25.0 25.0-29.9 30.0-34.9 35.0-39.9 >=40.0 *p*-values**

**(*n*=3,915) (*n*=11,144) (*n*=14,174) (*n*=8,023) (*n*=1,298)**

*Biological factors*

Female 48.6% 48.0% 48.7% 48.9% 48.8% *p*=0.77

Birthweight in grams^a^ 3012 3017 3010 2993 2927 *p*<0.01

(*SD*) (415) (415) (426) (458) (522)

Low birthweight 8.8% 8.5% 9.1% 11.0% 14.0% *p*<0.01

Preterm birth^a^ 5.2% 4.6% 5.2% 6.5% 9.6% *p<*0.01

Parity *p*<0.01

1 (no older siblings) 70.8% 56.7% 41.4% 33.1% 35.0%

2 25.5% 33.3% 41.9% 40.7% 34.1%

3>= 3.7% 10.0% 16.6% 26.1% 30.9%

*Socioeconomic factors*

Maternal educational attainment^a^ *p<*0.01

High school or less 65.2% 35.0% 24.6% 29.0% 33.4%

Two-year college 28.5% 39.0% 43.4% 44.9% 44.8%

or vocational school

Four-year college or 6.4% 26.0% 32.0% 26.1% 21.9%

higher

Paternal educational attainment^a^ *p*<0.01

High school or less 64.4% 40.2% 31.8% 34.1% 35.5%

Two-year college 19.1% 19.3% 18.6% 17.0% 16.6%

or vocational school

Four-year college or 16.4% 40.5% 49.7% 49.0% 47.9%

higher

Maternal employment status^a^

Employed full-time 34.2% 41.1% 35.8% 33.1% 32.1% *p*<0.01

Maternal smoking status^a^

Smoker 16.1% 7.2% 5.2% 5.5% 6.2% *p*<0.01

Paternal age^a^ 26.2 30.3 34.1 37.9 41.4 *p<*0.01

(*SD*) (4.8) (4.2) (4.0) (4.3) (5.0)

Family income^a^ 396 537 615 667 730 *p*<0.01

in JPY10,000 (*SD*) (210) (251) (300) (341) (451)

^a^ 7 missing cases for birthweight, 7 missing cases for preterm birth, 915 missing cases for breastfeeding duration, 173 missing cases for maternal educational attainment, 759 missing cases for paternal educational attainment, 205 missing cases for maternal employment status, 110 missing cases for maternal smoking status, 655 missing cases for paternal age, and 3453 missing cases for family income.
