# Supplementary material for: Association of maternal age with child health: A Japanese longitudinal study
Source: PLoS One. 2017 Feb 24;12(2):e0172544. doi: 10.1371/journal.pone.0172544 (PMC5325269; doi:10.1371/journal.pone.0172544)
Supplement: S2 Table — (DOC) [file pone.0172544.s002.doc]

S2 Table. Unadjusted, adjusted, and mediator-adjusted odds ratios with 95% confidence intervals and trend test results for associations of maternal age with unintentional injuries at 18 months of child age and hospital admissions at 18 and 66 months in the 2001 cohort (nparity1=22,967 and nparity2=17,119) and with unintentional injuries and hospital admissions at 18 months in the 2010 cohort (nparity1=18,079 and nparity2=14,365), stratified by maternal parity 1 and 2.

**Parity 1 Parity 2**

**Unadjusted Adjusted^a^ Mediator-adjusted^b^ *p*-trend Unadjusted Adjusted^a^ Mediator-adjusted^b^ *p*-trend**

2001 Cohort

Unintentional injuries

at 18 months *p*<0.01 *p*<0.01

<25.0 1.00 [reference] 1.00 [reference] 1.00 [reference] 1.00 [reference] 1.00 [reference] 1.00 [reference]

25.0-29.9 0.86 [0.78,0.94] 0.83 [0.73,0.94] 0.83 [0.74,0.94] 1.00 [0.84,1.18] 0.93 [0.75,1.15] 0.92 [0.75,1.14]

30.0-34.9 0.82 [0.74,0.91] 0.82 [0.72,0.94] 0.83 [0.72,0.95] 0.87 [0.74,1.03] 0.82 [0.66,1.03] 0.81 [0.65,1.01]

35.0-39.9 0.65 [0.56,0.75] 0.67 [0.56,0.81] 0.67 [0.56,0.81] 0.76 [0.63,0.91] 0.71 [0.56,0.92] 0.71 [0.55,0.91]

>=40.0 0.74 [0.52,1.05] 0.64 [0.43,0.96] 0.63 [0.42,0.94] 0.75 [0.52,1.08] 0.74 [0.48,1.13] 0.73 [0.47,1.12]

Hospital admission

at 18 months *p*=0.35 *p*=0.05

<25.0 1.00 [reference] 1.00 [reference] 1.00 [reference] 1.00 [reference] 1.00 [reference] 1.00 [reference]

25.0-29.9 0.91 [0.81,1.01] 0.93 [0.81,1.08] 0.93 [0.80,1.08] 0.91 [0.76,1.08] 0.99 [0.79,1.23] 0.99 [0.80,1.23]

30.0-34.9 0.82 [0.72,0.93] 0.88 [0.74,1.05] 0.88 [0.74,1.04] 0.84 [0.71,1.00] 0.98 [0.78,1.23] 0.98 [0.78,1.23]

35.0-39.9 0.92 [0.76,1.12] 1.00 [0.79,1.28] 1.00 [0.78,1.27] 0.62 [0.50,0.77] 0.75 [0.57,0.99] 0.75 [0.57,0.99]

>=40.0 0.75 [0.46,1.23] 0.76 [0.42,1.37] 0.76 [0.42,1.37] 0.77 [0.50,1.20] 0.88 [0.52,1.48] 0.87 [0.52,1.48]

Hospital admission

at 66 months *p*=0.90 *p*=0.23

<25.0 1.00 [reference] 1.00 [reference] 1.00 [reference] 1.00 [reference] 1.00 [reference] 1.00 [reference]

25.0-29.9 0.85 [0.72,1.01] 0.91 [0.73,1.14] 0.91 [0.73,1.13] 0.82 [0.59,1.13] 0.90 [0.59,1.35] 0.90 [0.60,1.36]

30.0-34.9 0.91 [0.76,1.09] 0.99 [0.77,1.28] 0.98 [0.76,1.26] 0.81 [0.59,1.11] 0.97 [0.63,1.50] 0.98 [0.63,1.51]

35.0-39.9 0.86 [0.65,1.14] 0.95 [0.66,1.36] 0.92 [0.64,1.32] 0.88 [0.61,1.27] 1.17 [0.72,1.91] 1.17 [0.72,1.91]

>=40.0 0.90 [0.47,1.73] 0.90 [0.41,1.97] 0.86 [0.39,1.89] 0.78 [0.36,1.69] 1.09 [0.46,2.55] 1.08 [0.46,2.54]

2010 Cohort

Unintentional injuries

at 18 months *p<*0.01 *p=*0.02

<25.0 1.00 [reference] 1.00 [reference] 1.00 [reference] 1.00 [reference] 1.00 [reference] 1.00 [reference]

25.0-29.9 0.94 [0.83,1.07] 0.73 [0.62,0.87] 0.73 [0.62,0.87] 1.14 [0.93,1.39] 0.95 [0.74,1.24] 0.95 [0.73,1.23]

30.0-34.9 0.83 [0.73,0.94] 0.62 [0.52,0.75] 0.63 [0.52,0.75] 1.15 [0.95,1.39] 0.92 [0.70,1.21] 0.92 [0.70,1.20]

35.0-39.9 0.76 [0.66,0.88] 0.61 [0.49,0.74] 0.61 [0.50,0.75] 0.97 [0.79,1.18] 0.77 [0.58,1.03] 0.77 [0.58,1.03]

>=40.0 0.86 [0.66,1.10] 0.63 [0.46,0.86] 0.64 [0.47,0.87] 1.03 [0.76,1.39] 0.84 [0.57,1.23] 0.85 [0.58,1.24]

Hospital admission

at 18 months *p*=0.52 *p*=0.37

<25.0 1.00 [reference] 1.00 [reference] 1.00 [reference] 1.00 [reference] 1.00 [reference] 1.00 [reference]

25.0-29.9 0.83 [0.70,0.98] 0.93 [0.74,1.17] 0.92 [0.73,1.16] 0.97 [0.76,1.24] 0.95 [0.70,1.29] 0.96 [0.71,1.31]

30.0-34.9 0.81 [0.68,0.96] 0.95 [0.74,1.22] 0.94 [0.73,1.21] 0.86 [0.68,1.08] 0.92 [0.67,1.27] 0.94 [0.68,1.30]

35.0-39.9 0.75 [0.61,0.92] 0.92 [0.69,1.23] 0.89 [0.67,1.19] 0.85 [0.66,1.08] 0.89 [0.63,1.26] 0.90 [0.63,1.27]

>=40.0 0.66 [0.45,0.97] 0.90 [0.56,1.43] 0.85 [0.53,1.35] 0.80 [0.55,1.17] 0.88 [0.55,1.41] 0.84 [0.53,1.35]

^a^ Adjusted for maternal and paternal education, maternal smoking status, maternal employment status one year prior to delivery (employed full-time or not), household income, sex of the child, maternal parity, and paternal age.

^b^ Adjusted for preterm births and birthweight in addition to confounders included in the adjusted model.
